# Using a trait-based approach to understand the efficiency of a selective device in a multispecific fishery

**DOI:** 10.1038/s41598-019-47117-4

**Published:** 2019-08-28

**Authors:** Maud Mouchet, Manon Poirson, Fabien Morandeau, Camille Vogel, Sonia Méhault, Dorothée Kopp

**Affiliations:** 1 UMR 7204 MNHN-SU-CNRS Centre d’Ecologie et des Sciences de la Conservation, CP135, 43 rue Buffon, 75005 Paris, France; 2 IFREMER, Unité de Sciences et Technologies Halieutiques, Laboratoire de Technologie et Biologie Halieutique, 8 rue François Toullec, F-56100 Lorient, France; 3 IFREMER, Department of Biological Resources and Environment/Fisheries Science for the English Channel and North Sea/Fisheries Resources Laboratory, Avenue du Général de Gaulle, 14520 Port-en-Bessin-Huppain, France

**Keywords:** Biodiversity, Environmental impact, Marine biology

## Abstract

Improving the selectivity of a fishing gear is one technical management measure to significantly reduce by-catch of non-commercial species or undersized individuals. The efficiency of selective device is mainly estimated by comparing species composition, the biomass and length spectrum of caught individuals and escapees while the functional traits of species are rarely accounted for. Using an innovative technical device to reduce catches of undersized individuals in a multispecific bottom trawl fishery in the Bay of Biscay, namely a T90 mesh cylinder, we measured functional traits on both caught and escaped individuals of 18 species. Using a Principal Component Analysis and K-means partitioning, we clustered species into 6 groups illustrating 6 different locomotion strategies. We identified functional traits related to body size, visual ability and locomotion, differing between caught individuals and escapees using Linear Mixed-effects Models. As expected, escapees were smaller on average but also tended to be more streamlined, with a high position of the eyes and fin features characteristic of manoeuvrability and propulsion. Here, we present how a trait-based approach can shed light on the biological characteristics influencing the efficiency of selective devices.

## Introduction

Multispecific fisheries using trawl gears generate discards through the capture of unwanted species, individuals below the minimum conservation reference size (MCRS)^1^ or because of the poor state of caught individuals^2^. To avoid or reduce the discard phenomenon, local, national and European authorities have implemented technical measures such as minimum mesh size^3^ or incentives to make fishing gears more selective by obligating the landing of the total catch of the regulated commercial species (under TAC - total allowable catch, and quota) so that these unwanted catches can be accounted for (Official Journal of the European Union 12/28/2013). Such constraints encouraged fishermen and gear technologists to develop a wide variety of trawl selective devices to select either species or individuals during the fishing operation, based on species or length criteria, namely inter- and intra- specific selectivity.

Inter-specific selectivity relies on differences of morphological features or behaviour between species. To let unwanted species escape from the gear, specific designs of the net, mesh or grids are to be adapted to their morphology^4–6^. The position and configuration of a selective device in the gear may also be adapted to the specific behaviour or swimming capacity of by-catch species^7–9^. When selectivity relies on body size, the mesh size may be determined by the MCRS^10^, although fitting the selection curve is often challenging^11^, especially due to variability of fish condition^12^, fish behaviour^13^ or fish contact probability with the selective device^9^. Recent developments in the understanding of selectivity suggest using the girth and the shape of the cross-section^14–16^, and in relation with the mesh shape and opening angle to predict size selectivity^17,18^.

Functional traits, i.e. traits revealing the links of an individual with its environment and ecosystem processes, have been extensively used to assess species niche, biotic interactions or environmental constraints. They are defined as individual characteristics (e.g. morphological, physiological, behavioural) “which impact fitness indirectly via their effects on growth, reproduction and survival, the three components of individual performance” (definition from^19^, see also references therein). Therefore, functional traits are defined to estimate the role of an individual in an ecological process or its response to its environment and allow a more predictive assessment of the effect of improving selectivity on community ecology and ecosystem functioning. Among all possible traits, mainly biomass, body length and/or girth and/or cross-section are applied to gear selectivity (see^15,20–23^), though the use of more traits could be more informative. One of the few studies investigating multiple traits in fisheries science highlighted correlative links between fish functional traits and several metiers (a given metier groups fishing operations based on their similarity in the fishing gear used, the species targeted, the geographical location and/or fishing season)^24^. But none, so far, has explored the links between several functional traits and selectivity.

In the Bay of Biscay, a wide diversity of marine species, encompassing various morphologies and behaviours, is available to the bottom trawl fishery^25^. Although several selective devices have been developed and tested^26^, implementing devices that efficiently reduce unwanted catch and by-catch remains challenging. Therefore, we propose to test whether functional traits of fish and cephalopod individuals caught during the sea trials of a T90 cylinder inserted in the extension of the trawl in the Bay of Biscay, can explain their response to the selective device. The T90 mesh based techniques, i.e. a diamond mesh turned 90° and remaining wide open throughout the fishing process, was first introduced in the early 1990s in the Baltic Sea^27^. It was later tested in the Bay of Biscay^28^ and in other European ecoregions^29,30^. Compared to similar diamond mesh sizes, enhanced selectivity in the codend is found for roundfish with T90 netting whereas it decreases selectivity for flatfish like plaice. So far, T90 meshes were mainly tested in the codend^29,31^ while other parts from the trawl might be relevant to increase fishing gear selectivity. Here, we applied a trait-based approach to understand the efficiency of a T90 cylinder in a two-step process: i) identifying if escapees and caught individuals have different functional profiles (i.e. combinations of trait values) and ii) identifying which traits significantly differ between the two groups of individuals (also called fractions).

## Results and Discussion

A total of 535 individuals, belonging to 18 species, were collected for the trait-based approach, i.e. 302 individuals caught inside the trawl (in 16 out of 21 hauls) and 233 escaped (in 17 out of 21 hauls) (Table 1). Fifteen species out of 18 were common to both fractions, even if these 15 species were not captured in every haul. Rays and plaices were exclusively caught in the trawl (i.e. found in the codend, respectively in 4 and 7 hauls) while all lesser-spotted dogfishes escaped (i.e. found exclusively in the cover and in a single haul).

**Table 1:** List of species encountered during the testing of the selective device.

Species			Number of individuals	
Scientific name	Common name	Code	Cover	Codend
*Engraulis encrasicolus*	European anchovy	ANCH	30	15
*Dicentrarchus labrax*	European seabass	BASS	15	21
*Trachurus trachurus*	Horse mackerel	HMAC	16	15
*Conger conger*	European conger	CONG	5	6
*Loligo sp*.	Squid	SQUI	4	11
*Spondyliosoma cantharus*	Black seabream	BREA	15	33
*Chelidonichthys cuculus*	Red gurnard	GUNA	4	24
*Scomber scombrus*	Atlantic mackerel	AMAC	16	7
*Merlangius merlangus*	Whiting	WHIT	7	6
*Merluccius merluccius*	European hake	HAKE	22	29
*Mugil spp*	Mullet	MULL	15	3
*Pleuronectes platessa*	European plaice	PLAI	—	10
*Raja sp*.	Ray	RAY	—	13
*Mullus surmuletus*	Red mullet	RMUL	20	28
*Scyliorhinus canicula*	Lesser-spotted dogfish	DOG	4	—
*Sepia officinalis*	Cuttlefish	CUTL	14	27
*Solea solea*	Sole	SOLE	24	37
*Micromesistius poutassou*	Blue whiting	BWHI	22	17

The number of individuals in the table refers to the number of individuals used for measuring functional traits. “Codend” = caught individuals; “Cover” = escapees.

**Figure 1:**
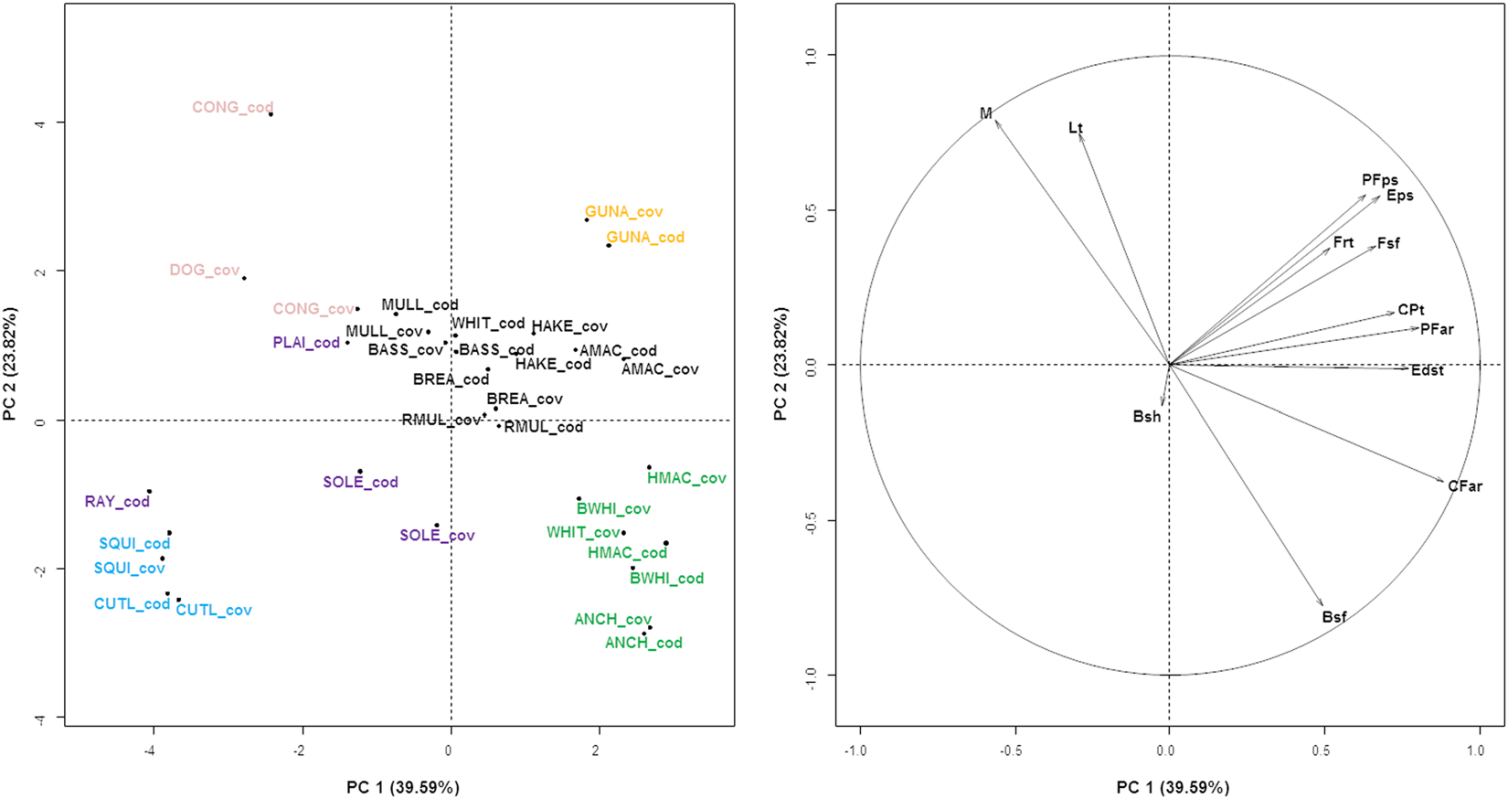
Identification of six functional profiles using a Principal Component Analysis and K-means partitioning. Abbreviations for species names and functional trait are reported in Tables 1, 2, respectively. “_cod” and “_cov” discriminate caught individuals (in the codend) and escapees (in the cover), respectively. The circle indicates the correlation circle of the PCA results on variables.

The first 3 axes of the PCA carried out for all species and fractions summarized 75.44% of the total inertia. PCA and K-means analyses revealed 6 functional profiles (Silhouette index = 0.87) (Fig. 1). Four profiles out of 6 corresponded to very specific morphologies and swimming strategies (only traits discriminating each cluster and identified by the *catdes* function are mentioned here): i) flatfishes (soles, SOLE, and plaices, PLAI) and rays (RAY) characterized by relatively small eyes (low values of Edst) and a high body surface (Bsh); ii) conger (CONG) and lesser-spotted dogfish (DOG), elongated species with no proper caudal fin (low average values of CFar) and a body mass higher (high average values of Lt and M) than most species in our pool; iii) cephalopods (squid, SQUI, and cuttlefish, CUTL) had low values for most traits [body transversal shape (Bsh), caudal peduncle throttling (CPt), relative eye size (Edst), aspect ratios of the caudal fin (CFar), and of pectoral fins (PFar), relative surface of fins (Fsf), pectoral fin position (PFps) and eye position (Eps)]; iv) Red gurnard (GUNA) with its distinctive characteristics, i.e. down positioned pectoral fins (PFps), high fin surface (Frt and Fsf) and upward position of the eyes (Eps). The last 2 groups clustered mostly bentho-pelagic species: v) anchovy (ANCH), whiting (WHIT, escapees), blue whiting (BWHI) and horse mackerel (HMAC) characterized by, on average, higher values of the body transversal surface (Bsf), the relative size of the eyes (Edst) and the aspect ratios of the caudal fin (CFar) and the pectoral fins (PFar) but lower values of total length (Lt) and biomass (M); vi) Seabass (BASS), seabream (BREA), hake (HAKE), mullet (MULL), red mullet (RMUL), Atlantic mackerel (AMAC) and whiting (WHIT, caught) discriminated by high values of the position of pectoral fins (PFps), the eye position (Eps) and relative size (Edst) and the caudal peduncle throttling (CPt) but a low body surface (Bsf). Apart from whiting, escapees and caught individuals from the same species were clustered in the same group, suggesting that the intraspecific variability across the two fractions is far lower than the interspecific variability. One strategy to improve the comparison of intra- and inter-specific variabilities and allow a finer discrimination of functional profiles of escapees and caught individuals in future studies could be to increase significantly the number of individuals sampled for the functional characterization, per species, per fraction and throughout space and seasons. In the case of whiting, escapees differed from caught individuals, regarding total length (Lt), biomass (M), eye size (Edst), body surface (Bsf), caudal fin aspect ratio (CFar) and fin surface (Fsf). Escapees seemed, on average, heavier than caught individuals but their length was still lower while their fin surface was much higher. This might be related to a high muscular mass of the small whiting relatively to their size, enabling their escapement.

Although, the PCA did not discriminate escapees from caught individuals, it highlighted some groups of species among which some species are known to be able to escape (or not)^32^, thereby highlighting combinations of trait values that might help escaping (or not). Our approach shows that horse mackerel benefits from high values for functional traits responding to selectivity [body surface (Bsf), aspect ratio of the caudal fin (CFar), aspect ratio (PFar) and position (PFps) of pectoral fins], while cuttlefish has low values for these traits. These results are consistent with the selectivity curves from Kopp *et al*.^32^ which showed that horse mackerel were able to escape while cuttlefish could not. Likewise, congers and dogfishes might be able to force their way out due to undulatory movements and a muscular body^33,34^, helped by its fusiform shape in the case of conger, until their body section far exceeds the mesh size. Conversely, the swimming mode based on propulsion of red gurnards, rays and cephalopods, and the lower sustained swimming speeds and endurance of flatfishes^35–37^ may be inefficient once in the trawl. This disadvantage may be strengthened by an inadequate visual acuity due to a small eye and/or an eye positioned in a way that the orientation of the visual field compromises the ability to detect a mesh. However, previous findings suggest that some flatfishes, like sole, are able to escape through selective devices^32,38,39^. Here, we did not consider behavioural characteristics (e.g. schooling behaviour, active swimming) nor the contact probability of the species with the selective device that may modulate the morphological ability to escape. In addition, and to our knowledge, no functional trait was defined specifically for cephalopods, so we used functional traits established for fishes and did not consider the tentacles of squids or the fin surrounding the mantle of cuttlefish. Fish functional traits characterizing body shape and size as well as visual acuity are likely relevant but further investigations on cephalopods’ specific functional traits, especially related to locomotion, may be interesting to improve our understanding of their ability to escape a selective device. Finally, we cannot exclude that our findings are partly biased for the species for which traits related to fins tended to zero, i.e the caudal fin of congers, rays and lesser-spotted dogfishes and/or the pectoral fins of flatfishes and rays, because the fins of these species could not be clearly delineated.

**Figure 2:**
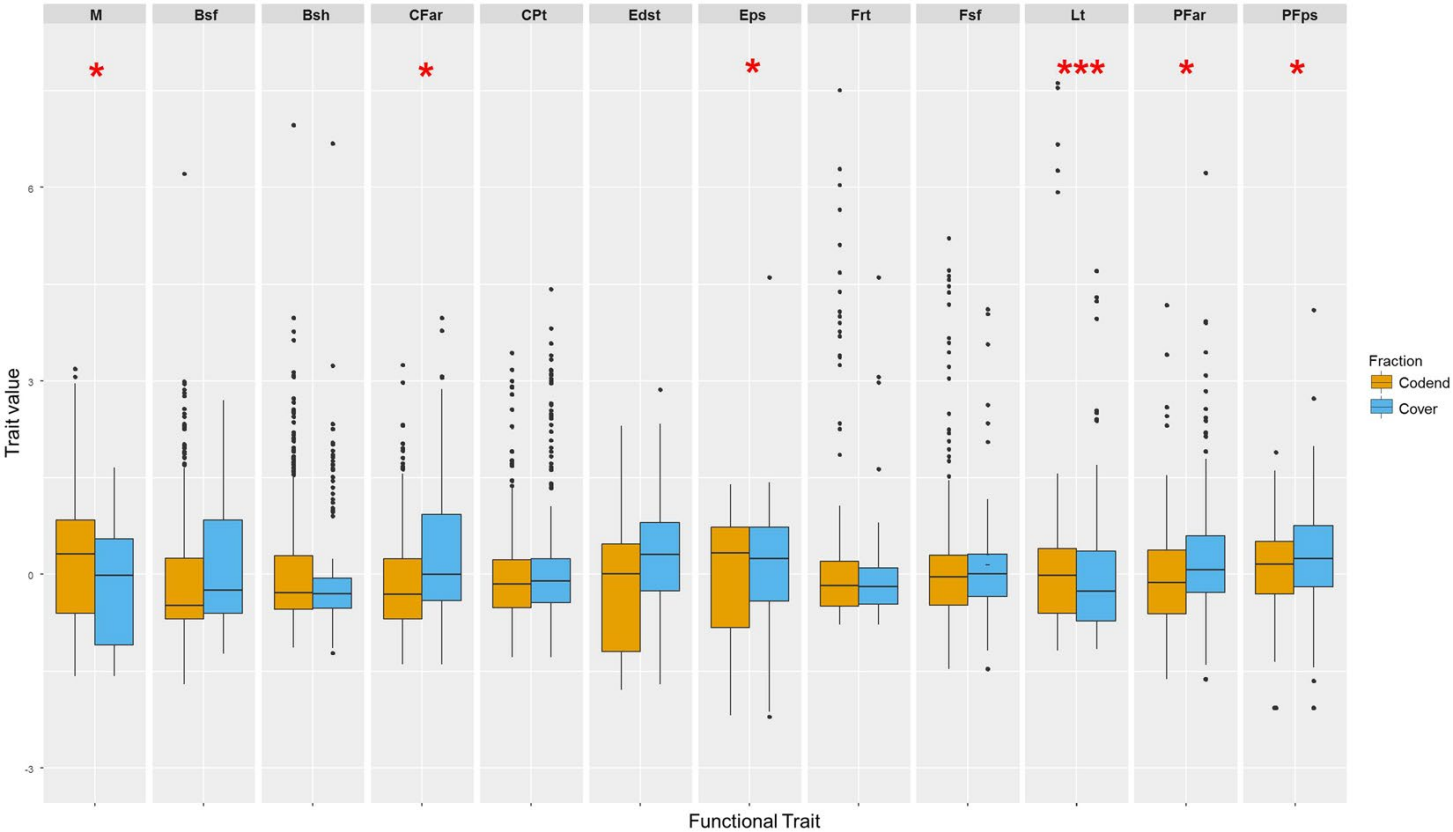
Differences in the distribution of functional trait values between escapees (in the cover, blue boxes) and caught individuals (in the codend, yellow boxes). Significant levels are given by the p-value associated to the estimate of the fixed effect (i.e. the fraction) in the Linear Mixed-effects Model. “ ”p < 0.1, “*”p ≤ 0.05, “**”p ≤ 0.01, “***”p ≤ 0.001, “****”p ≤ 0.0001. The acronyms are defined in Table 2.

Using linear mixed-effects models for each functional trait, we found significant differences among both fractions for average values of: the total length (Lt), the standardized biomass (M), the eye position (Eps), the aspect ratio of the caudal fin (CFar), the position (PFps) and the aspect ratio (PFar) of the pectoral fin (Fig. 2). Specifically, the average values of Eps, PFps, PFar and CFar were higher for escapees than for caught individuals while the average values of Lt and M were lower for escapees. Interestingly, we found no significant difference for body transversal shape (Bsh) and body transversal surface (Bsf) that reflect, respectively, cross-section and girth, two parameters used in selectivity studies. Excluding cephalopods from the models gave similar results (results not shown here), suggesting that potential biases related to the use of fish functional traits to characterize cephalopods do not change our findings.

Overall, our results suggest that traits related to body size, visual acuity and locomotion are involved in the ability to escape. As expected, average biomass and average total length of the caught individuals are significantly higher than those of escapees. Larger individuals are likely to be mechanically restrained by the mesh size. The relative position of the eye might play a role in visual acuity, together with the absolute eye size^40,41^, and is expected to contribute to the detection of the meshes. This finding could be used to improve future selective devices with, for example, coloured nets^42,43^ or lights^44^ that stimulate fish escapement through visual stimuli. Species, and in particular streamlined species like horse mackerel, characterized by an upward position of the pectoral fins tend to be more efficient regarding speed and manoeuvrability^45,46^, which increases their chance of escaping the gear. The aspect ratios of the pectoral and the caudal fins are both involved in propulsion, more specifically burst mode using crescent-like caudal fins, and steering mechanisms^47,48^. This could be used to test future selective devices. For instance, for species with low manoeuvrability such as cephalopods, T90 or square meshes^49,50^ could be tested in the codend to allow the escape of small individuals that are not efficient at escaping through the cylinder. This T90 cylinder mounted in the extension part of a coastal otter trawl provides fishermen with a selective device relevant to reduce discards. The device appears especially efficient when by-catch is made of high proportion of pelagic species displaying high maneuverability and visual acuity, as demonstrated for horse mackerel, anchovy or blue whiting in our experiment. Such a selective device may therefore be of interest for other multispecies demersal fisheries facing by-catch of anchovy, sardine and sprat (in the Bay of Biscay), and herring (in the Channel and North Sea areas).

Using a trait-based approach, we were able to cluster species into 6 functional trait profiles and to identify several functional traits that significantly differed among the 2 fractions of individuals (caught and escaped). Our study highlights the complexity of the relationship between body size, morphology and selectivity and the need for a better integration of the functional, physiological and behavioural response of species to a selective device. Such complexity needs to be further examined to document trait variability according to seasonality, ontogenetic changes, geographical position, differences in species diversity among hauls, etc. Extending such approach to other fisheries context should provide fisherman and fisheries stakeholders with a toolbox to increase their understanding of the mechanisms underpinning selectivity and to help them choosing the appropriate selective device for a given metier. One limitation to the integration of a trait-based approach might be the measurements of traits for numerous species but it should become more and more feasible with the development of biological trait databases (FishBase^51^, WoRMS^52^). Finally, by selecting individuals and species on specific biological traits, selectivity devices inserted in a trawl body might contribute to remove particular combinations of traits from the ecosystem thereby favouring other combinations of traits. Therefore introducing a more complete trait-based approach to selectivity studies should help foreseeing the effects of selectivity on community structure and ecological functions.

## Methods

### Selectivity experiment

Fish species and cephalopods were sampled in June 2016 in the fishing grounds of the Bay of Bourgneuf (Bay of Biscay, France) during the sea trials of a selective device, i.e. a T90 extension piece. The sea trials were authorised by the Ministère de l'Écologie, du Développement durable et de l'Énergie under permission number 2016/930461/FUSION/0001. The T90 extension piece was mounted on the single otter trawl of a commercial trawler and the sea trials were carried out following normal commercial practices during daytime at the depth and location the trawler would normally fish. Only the tow duration was shortened to one hour for an optimal manipulation of the cover when hauling back the trawl. The extension piece had a netting orientation turned at 90° (T90) with a 72 mesh circumference and 40 mesh length polyethylene (PE). Its selective performance was estimated using the covered codend method, held open by kites^53^. The cover was made of polyamide netting with a 20 mm nominal mesh size, a circumference of 1370 meshes and a length twice as long as the extension piece and codend combined. Fish were prevented from escaping the anterior and posterior part of the extension piece by: i) a flapper (70 mm mesh) in the anterior part, ii) an overlapping net (100 mm mesh) used as connection with the inner bag in the posterior part. To avoid escape from the codend, a fine mesh inner bag of 37.2 ± 0.8 mm mesh size (20 mm nominal) was inserted there. Prior to sea trials, the kite cover and the selective device were tested and validated in the flume tank at IFREMER Lorient^54^, using a half-scale model. For an extensive description of the selective device, refer to Kopp *et al*.^32^ and Fig. S1 in the Supplementary Information. A total of 21 hauls were performed at a mean depth of 11 m (±4 m), with an average vessel speed of 3.5 knots. After each haul, the total catches from the codend (i.e. the fine mesh inner bag) and from the cover were sorted separately and by species. Individuals found in the codend were considered as caught individuals whereas individuals found in the cover were considered as escapees.

### Ecological and biological characterisation of sampled individuals and species

To test whether the ecological and biological traits of species might play a role in species ability to escape through the selective device, we calculated 12 standardized traits, i.e. functional traits (Table 2) related to locomotion and visual acuity following Villéger *et al*.^41,55^. The calculation of functional traits is based on 14 ecomorphological features measured on each individual sampled (Supplementary Information: Fig. S2). As in Villéger *et al*.^41,55^, we distinguished ecomorphological features from functional traits. In our analyses, we included only functional traits in order to: i) avoid a circular reasoning (since functional traits are calculated from ecomorphological features) and ii) allow for a robust size-independent interpretation (since most ecomorphological features are susceptible to be correlated with body size, i.e. length or biomass). Small individuals are more likely to exhibit lower values for a given ecomorphological feature. On the contrary, functional traits were constructed to be as size-independent as possible (except for length and biomass, obviously) and are therefore appropriate to go beyond a simplistic size-based approach of selectivity. Several individuals were collected randomly for the measurement of ecomorphological features, specifically we collected from 3 to 37 individuals of each species in each fraction (i.e. caught individuals in the codend and escapees in the cover, see Table 1). Functional traits were calculated for each individual and then averaged at the species level but for each fraction separately.

**Table 2:** List of functional traits (from^41,55^). The abbreviations mentioned in the quantification of functional traits refer to the ecomorphological features used and are presented in Supplementary Information Fig. S2.

Functional trait	Code	Quantification
Eye size	Edst	$\frac{Ed}{Hd}$
Eye position	Eps	$\frac{Eh}{Hd}$
Body transversal shape	Bsh	$\frac{Bd}{Bw}$
Body transversal surface	Bsf	$\frac{\mathrm{log}(\frac{\pi }{4}\times Bw\times Bd)+1}{log(B+1)}$
Pectoral fin position	PFps	$\frac{PFi}{PFd}$
Aspect ratio of the pectoral fin	PFar	$\frac{PF{l}^{2}}{PFs}$
Caudal peduncle throttling	CPt	$\frac{CFd}{CPd}$
Aspect ratio of the caudal fin	CFar	$\frac{CFd}{CFs}$
Fins surface ratio	Frt	$\frac{2\times PFs}{CFs}$
Fins surface to body size ratio	Fsf	$\frac{(2\times PFs)+CFs}{\frac{\pi }{4}\times Bw\times Bd}$
Biomass	M	$\mathrm{log}(B+1)$
Total length	Lt	Absolute Lt

Bd: maximal body depth; Bw: maximal body width; CFd: maximal caudal fin depth; CFs: caudal fin surface; CPd: peduncle minimal depth; Ed: eye diameter; Eh: eye position; Hd: head depth; Lt: total length; PFd: body height at the pectoral fin insertion; PFi: position of the pectoral fin; PFl: maximal fin length; PFs: pectoral fin surface. B: body weight. Lt is considered as an ecomorphological feature as well as a functional trait. All traits are dimensionless, excepted M (in grams) and Lt (in millimetres).

### Data analysis

To identify the functional profiles (i.e. combinations of trait values) differing between the two fractions of individuals (caught and escaped), we performed a Principal Component Analysis (PCA) on the species-traits matrix. For a given species, we considered individuals from the codend (caught individuals) and the cover (escapees) as two distinct species matrices and we calculated the average trait value per species and per fraction. To objectively cluster species into groups, we used the K-means partitioning method on the coordinates of the species on the 8^th^ first principal components (8 being the minimal number of components required, in our case, so that the quality of representation of each species on the PCA, i.e. cos^2^, is equal or superior to 0.8). Then we used the Silhouette index to determine the optimal number of clusters. Finally, we used the *catdes* function (FactoMineR package) to identify which (levels or modalities) of traits are significantly associated with each cluster. This function is based on the v-test that tests whether the mean value of a group of observations (i.e. species) for a given variable (e.g. a functional trait) significantly differs from the mean value of the whole population, all groups considered^56^. In a second step, we sought to identify the traits that differed between caught and escaped individuals. To that end, we tested for a significant difference in the measured functional traits between caught and escaped individuals, using a Linear Mixed-effects Model. In the models, the trait values were the response variable, the fraction (“codend” or “cover”) was the fixed effect and the species was integrated as a random effect because trait values of individuals of the same species in a given fraction are susceptible to be clustered. To remove a potential bias in the functional characterization of cephalopods due to the use of traits primarily designed for fishes, we also ran the models on fish individuals only. The sampling strategy aimed at looking into individual variability, therefore randomness arising from the different hauls was not considered here. The tests were performed with the *lmer* function (lme4 package). We further illustrated the comparison of mean trait values between fractions with boxplots. All analyses were performed under R (version 3.3.3^57^) using FactoMineR^58^, ggplot2^59^, ggsignif^60^, ggpubr^61^, cluster^62^, lme4^63^ and vegan^64^ packages.

## Supplementary information

Using a trait-based approach to understand the efficiency of a selective device in a multispecific fishery.

## References

[1] Kumar AB, Deepthi GR (2006). Trawling and by-catch: Implications on marine ecosystem. Curr. Sci..

[2] Catchpole TL, Frid CLJ, Gray TS (2005). Discards in North Sea fisheries: causes, consequences and solutions. Mar. Policy.

[3] EU. Concil regulation (EC) No 850/98 of 30 March 1998 for the conservation of fishery resources through technical measures for the protection of juveniles of marine organisms (1998).

[4] Aydin C, Tokac A, Aydin I, Erdogan U, Maktay B (2011). Species selectivity in the Eastern Mediterranean demersal trawl fishery using grids to reduce non-target species. J. Appl. Ichthyol..

[5] Broadhurst MK, Millar RB, Wooden MEL, Macbeth WG (2006). Optimising codend configuration in a multispecies demersal trawl fishery. Fish. Manag. Ecol..

[6] Herrmann B, Sistiaga M, Larsen RB, Nielsen KN, Grimaldo E (2013). Understanding sorting grid and codend size selectivity of Greenland halibut (Reinhardtius hippoglossoides). Fish. Res..

[7] Herrmann B (2015). Understanding the release efficiency of Atlantic cod (Gadus morhua) from trawls with a square mesh panel: effects of panel area, panel position, and stimulation of escape response. ICES J. Mar. Sci..

[8] Krag LA, Herrmann B, Feekings J, Lund HS, Karlsen JD (2016). Improving escape panel selectivity in Nephrops-directed fisheries by actively stimulating fish behavior. Can. J. Fish. Aquat. Sci..

[9] Santos J, Herrmann B, Otero P, Fernandez J, Pérez N (2016). Square mesh panels in demersal trawls: does lateral positioning enhance fish contact probability?. Aquat. Living Resour..

[10] Alzorriz N (2016). Questioning the effectiveness of technical measures implemented by the Basque bottom otter trawl fleet: Implications under the EU landing obligation. Fish. Res..

[11] Millar RB (2010). Reliability of size-selectivity estimates from paired-trawl and covered-codend experiments. ICES J. Mar. Sci..

[12] Ozbilgin H, Tosunoglu Z, Tokac A, Metin G (2011). Seasonal variation in the trawl codend selectivity of Red mullet (Mullus barbatus). Turk. J. Fish. Aquat. Sci..

[13] Krag LA, Madsen N, Karlsen JD (2009). A study of fish behaviour in the extension of a demersal trawl using a multi-compartment separator frame and SIT camera system. Fish. Res..

[14] Mendes B, Fonseca P, Campos A (2006). Relationships between opercular girth, maximum girth and total length of fish species caught in gillnet and trammel net selectivity surveys off the Portuguese coast. J. Appl. Ichthyol..

[15] Carol J, Garcia-Berthu E (2007). Gillnet selectivity and its relationship with body shape for eight freshwater fish species. J. Appl. Ichthyol..

[16] Jawad LA, McKenzie A, Al‐Noor SS (2009). Relationship between opercular girth, maximum girth and total length of fishes caught in gillnets in the estuarine and lower river sections of Shatt al Arab River (Basrah Province, Iraq). J. Appl. Ichthyol..

[17] Herrmann B (2009). Prediction of selectivity from morphological conditions: methodology and a case study on cod (Gadus morhua). Fish. Res..

[18] Tokac A (2016). Understanding the size selectivity of red mullet (Mullus barbatus) in Mediterranean trawl codends: A study based on fish morphology. Fish. Res..

[19] Violle C (2007). Let the concept of trait be functional!. Oikos.

[20] Clabaut C, Bunje PME, Salzburger W, Meyer A (2007). Geometric Morphometric Analyses Provide Evidence for the Adaptive Character of the Tanganyikan Cichlid Fish Radiations. Evolution.

[21] Díaz de Astarloa JM (2011). Morphological, morphometric, meristic and osteological evidence for two species of hake (Actinopterygii: Gadiformes: Merluccius) in Argentinean waters. J. Fish Biol..

[22] Rodríguez-Mendoza R, Muñoz M, Saborido-Rey F (2011). Ontogenetic allometry of the bluemouth, Helicolenus dactylopterus dactylopterus (Teleostei: Scorpaenidae), in the Northeast Atlantic and Mediterranean based on geometric morphometrics. Hydrobiologia.

[23] Tosunoğlu Z (2007). Trawl codend design (44 mm diamond PE mesh) and the effect on selectivity for Pagellus erythrinus and Pagellus acarne, two species with different morphometrics. J. Appl. Ichthyol..

[24] Koutsidi M, Tzanatos E, Machias A, Vassilopoulou V (2016). Fishing for function: the use of biological traits to evaluate the effects of multispecies fisheries on the functioning of fisheries assemblages. ICES J. Mar. Sci..

[25] Fauconnet L, Trenkel VM, Morandeau G, Caill-Milly N, Rochet MJ (2015). Characterizing catches taken by different gears as a step towards evaluating fishing pressure on fish communities. Fish. Res..

[26] Vogel C, Kopp D, Mehault S (2017). From discard ban to exemption: How can gear technology help reduce catches of undersized Nephrops and hake in the Bay of Biscay trawling fleet?. J. Environ. Manag..

[27] Moderhak W (1997). Determination of selectivity of cod codends made of netting turned through 90 degree. Bull. Sea Fish Inst..

[28] Meillat, M. & Morandeau, F. Hake selectivity - Summary of work aiming to improve the selectivity of *Nephrops*/hake bottom trawls. *IFREMER* Available at https://w3.ifremer.fr/archimer/doc/00347/45793/ (2001).

[29] Madsen N, Herrmann B, Frandsen RP, Krag LA (2012). Comparing selectivity of a standard and turned mesh T90 codend during towing and haul-back. Aquat. Living Resour..

[30] Tokac A (2014). Predictive models and comparison of the selectivity of standard (T0) and turned mesh (T90) codends for three species in the Eastern Mediterranean. Fish Res..

[31] Herrmann B, Wienbeck H, Moderhak W, Stepputtis D, Krag LA (2013). The influence of twine thickness, twine number and netting orientation on codend selectivity. Fish Res..

[32] Kopp D, Morandeau F, Mouchet MA, Vogel C, Méhault S (2018). What can be expected from a T90 extension piece to improve selectivity in bottom trawl mixed fisheries in the Bay of Biscay?. Fish. Sci..

[33] Grillner, S. & Kashin, S. On the generation and performance of swimming in fish. In: Herman R. M., Grillner, S., Stein P. & DGS (ed). *Neural Control of Locomotion*. Plenum, New York, pp 191–202 (1976).

[34] Lauder, G. V. & Tytell, E. D. Hydrodynamics of undulatory propulsion. In: Shadwick, R. E. & Lauder, G. V. (eds). *Fish Physiology: Fish Biomechanics*. Elsevier, pp 126–132 (1998).

[35] Ryer CH (2008). A review of flatfish behavior relative to trawls. Fish. Res..

[36] Bayse SM (2016). Could a T90 mesh codend improve selectivity in the Belgian beam trawl fishery?. Fish. Res..

[37] Winger PD, Walsh SJ, He P, Brown JA (2004). Simulating trawl herding in flatfish: the role of fish length in behaviour and swimming characteristics. ICES J. Mar. Sci..

[38] Krag LA, Herrmann B, Karlsen JD (2014). Inferring fish escape behaviour in trawls based on catch comparison data: Model development and evaluation based on data from Skagerrak, Denmark. PLoS One.

[39] Santos J, Herrmann B, Mieske B, Stepputtis D, Krumme U, Nilsson H (2016). Reducing flatfish bycatch in roundfish fisheries. Fish. Res..

[40] Piet GJ (1998). Ecomorphology of a size-structured tropical freshwater fish community. Environ. Biol. Fishes.

[41] Villéger S, Brosse S, Mouchet MA, Mouillot D, Vanni MJ (2017). Functional ecology of fish: current approaches and future challenges. Aquat. Sci..

[42] Glass CW, Wardle CS (1995). Studies on the use of visual stimuli to control fish escape from codends. II. The effect of a black tunnel on the reaction behaviour of fish in otter trawl codends. Fish. Res..

[43] Jones, E. G., Fryer, R., Kynoch, R. & Summerbell, K. Working Document: The influence of twine colour and contrast on the effectiveness of square mesh panels in a demersal whitefish trawl. In: 2. The reaction and behaviour of fish to visual components of fishing gears and the effect of catchability in survey and commercial situations. *ICES WGFTFB Working Paper* 100–109 (2004).

[44] Hannah RW, Lomeli MJM, Jones SA (2015). Tests of artificial light for bycatch reduction in an ocean shrimp (Pandalus jordani) trawl: Strong but opposite effects at the footrope and near the bycatch reduction device. Fish Res..

[45] Bellwood DR, Wainwright PC (2001). Locomotion in labrid fishes: implications for habitat use and cross-shelf biogeography on the Great Barrier Reef. Coral Reefs.

[46] Dumay O, Tari PS, Tomasini JA, Mouillot D (2004). Functional groups of lagoon fish species in Languedoc Roussillon, southern France. J. Fish Biol..

[47] Webb PW (1984). Body form, locomotion and foraging in aquatic vertebrates. Am. Zool..

[48] Fulton CJ, Bellwood DR, Wainwright PC (2001). The relationship between swimming ability and habitat use in wrasses (Labridae). Mar. Biol..

[49] Ordines F, Massutí E, Guijarro B, Mas R (2006). Diamond vs. square mesh codend in a multi-species trawl fishery of the western Mediterranean: effects on catch composition, yield, size selectivity and discards. Aquat. Living Resour..

[50] Sala A, Lucchetti A, Piccinetti C, Ferretti M (2008). Size selection by diamond- and square-mesh codends in multi-species Mediterranean demersal trawl fisheries. Fish Res..

[51] Froese, R. & Pauly, D. Editors. FishBase 2000: concepts, design and data sources. *ICLARM, Los Baños, Laguna, Philippines*. 344 p (2000).

[52] Horton, T. *et al*. World Register of Marine Species. Available from http://www.marinespecies.org at VLIZ (2018).

[53] Madsen N, Hansen KE, Moth-Poulsen T (2001). The kite cover: a new concept for covered codend selectivity studies. Fish. Res..

[54] Vincent B (2018). Lorient flume tank. Ifremer..

[55] Villéger S, Ramos Miranda J, Flores Hernandez D, Mouillot D (2010). Contrasted changes in taxonomic and functional diversity of tropical fish communities after habitat degradation. Ecol. Appl..

[56] Husson, F., Le, S. & Pages, J. Exploratory Multivariate Analysis by Example Using R. Chapman and Hall/CRC Press, Boca Raton, USA. 240 pp. (2010).

[57] R Core Team. R: A language and environment for statistical computing. R Foundation for Statistical Computing, Vienna, Austria, https://www.R-project.org/ (2017).

[58] Lê S, Josse J, Husson F (2008). FactoMineR: An R Package for Multivariate Analysis. J. Stat. Softw..

[59] Wickham, H. ggplot2: elegant graphics for data analysis. Springer-Verlag New York, http://ggplot2.org (2009).

[60] Ahlmann-Eltze, C. ggsignif: Significance Brackets for ‘ggplot2’. R package version 0.4.0, https://CRAN.R-project.org/package=ggsignif (2017).

[61] Kassambara, A. ggpubr: ‘ggplot2’ Based Publication Ready Plots. R package version 0.1.6, https://CRAN.R-project.org/package=ggpubr (2017).

[62] Maechler, M., Rousseeuw, P., Struyf, A., Hubert, M. & Hornik, K. cluster: cluster analysis basics and extensions. R package version 2.0.6 (2017).

[63] Bates D, Maechler M, Bolker B, Walker S (2015). Fitting Linear Mixed-Effects Models Using lme4. J. Stat. Softw..

[64] Oksanen, J. *et al*. vegan: community ecology Package. R package version 2.4-6, https://CRAN.R-project.org/package=vegan (2018).

