# Supplementary material for: Using a trait-based approach to understand the efficiency of a selective device in a multispecific fishery
Source: Sci Rep. 2019 Aug 28;9:12489. doi: 10.1038/s41598-019-47117-4 (PMC6713766; doi:10.1038/s41598-019-47117-4)
Supplement: Supplementary file 1 — Using a trait-based approach to understand the efficiency of a selective device in a multispecific fishery. [file 41598_2019_47117_MOESM1_ESM.docx]

**Supplementary Information**

**Using a trait-based approach to understand the efficiency of a selective device in a multispecific fishery.**

Maud Mouchet^1^*, Manon Poirson^1^, Fabien Morandeau^2^, Camille Vogel^3^, Sonia Méhault^2^ & Dorothée Kopp^2^

**^1^** UMR 7204 MNHN-SU-CNRS Centre d’Ecologie et des Sciences de la Conservation, CP135, 43 rue Buffon, 75005 Paris,  France; **^2^** IFREMER, Unité de Sciences et Technologies Halieutiques, Laboratoire de Technologie et Biologie Halieutique, 8 rue François Toullec, F-56100 Lorient, France ; **^3^** IFREMER, Department of Biological Resources and Environment/ Fisheries Science for the English Channel and North Sea/Fisheries Resources Laboratory, Avenue du Général de Gaulle, 14520 Port-en-Bessin-Huppain, France

* Contact author: Maud Mouchet, UMR 7204 MNHN-SU-CNRS Centre d’Ecologie et des Sciences de la Conservation, CP135, 43 rue Buffon, 75005 Paris, France. [maud.mouchet@mnhn.fr](mailto:maud.mouchet@mnhn.fr)

Figure S1. Diagram of the T90 extension piece mounted on the otter trawl and surrounded by a polyamide netting cover and a picture of the experimental device during the preliminary tests in the flume tank of IFREMER Lorient.


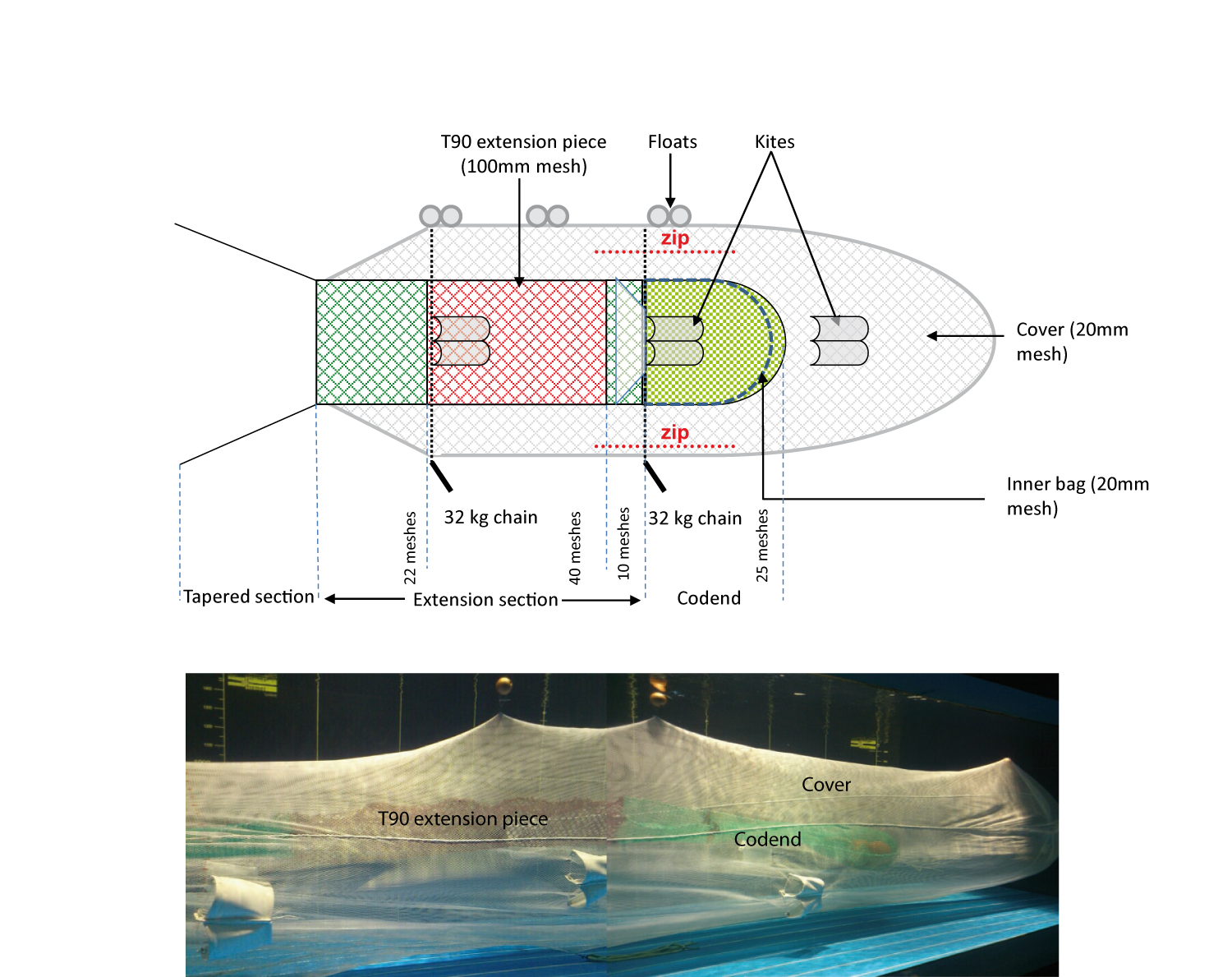


Figure S2. Ecomorphological features used to calculate functional traits in this trait-based approach to selectivity (from ^1,2,3^). Bd: maximal body depth; Bw: maximal body width; CFd: maximal caudal fin depth; CFs: caudal fin surface; CPd: peduncle minimal depth; Ed: eye diameter; Eh: eye position; Hd: head depth; Lt: total length; PFd: body height at the pectoral fin insertion; PFi: position of the pectoral fin; PFl: maximal fin length; PFs: pectoral fin surface. Body weight (B) was directly measured on individuals during the sea trials. Lt is considered as an ecomorphological feature as well as a functional trait. Lengths, heights and depths are measured in millimetres, surfaces in square millimetres and body weight in grams.


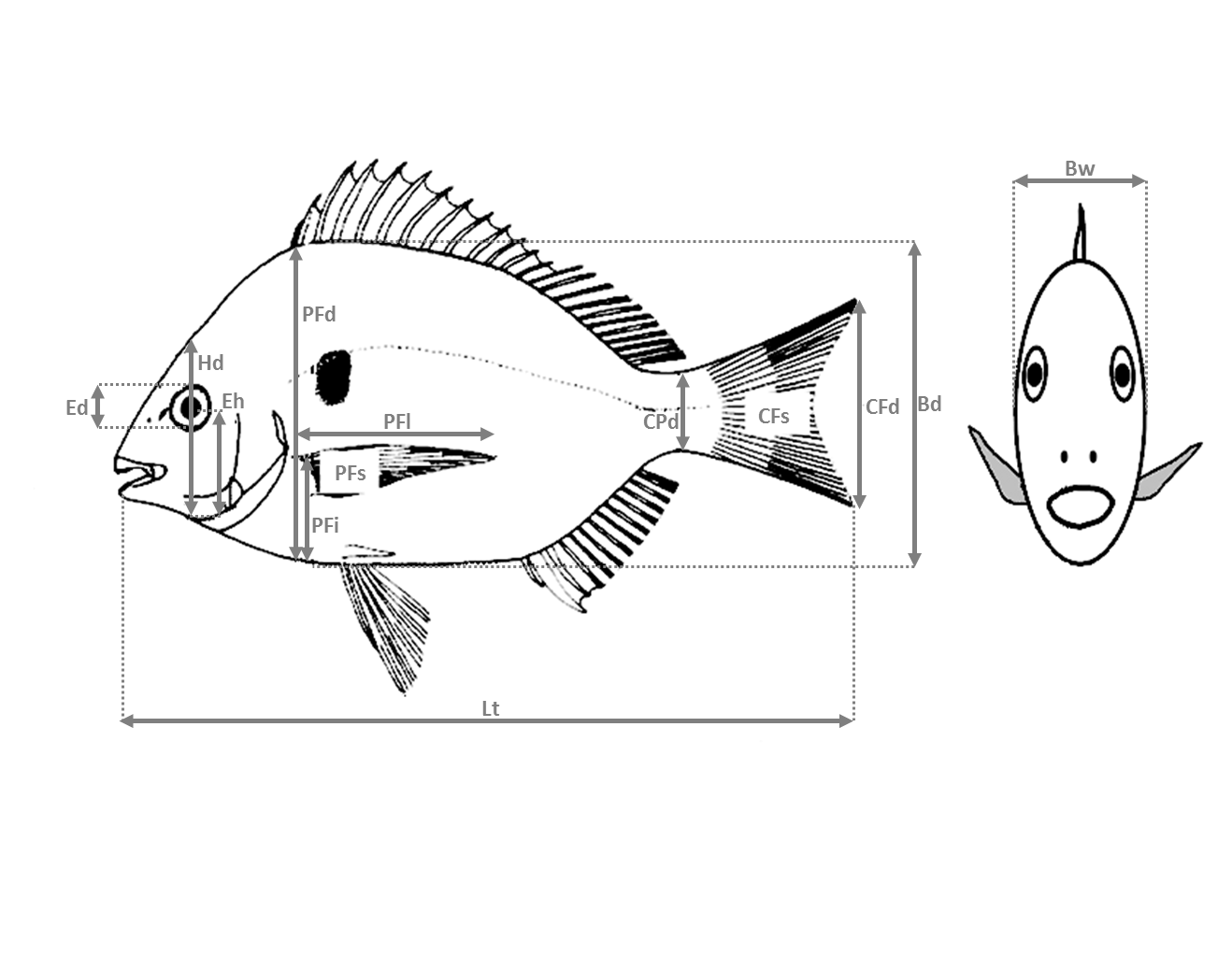


1. Mouchet, M. A., Burns, M. D. M., Garcia, A. M., Vieira, J. P. & Mouillot D. Invariant scaling relationship between functional dissimilarity and co-occurrence in fish assemblages of the Patos estuary (Brazil): environmental filtering consistently overshadows competitive exclusion. *Oikos* **122**, 247-257 (2013).

Villéger, S., Brosse, S., Mouchet, M. A., Mouillot, D. & Vanni M.J. Functional ecology of fish: current approaches and future challenges. *Aquat. Sci.* **79**, 783-801 (2017).

Villéger, S., Ramos Miranda, J., Flores Hernandez, D. & Mouillot D. Contrasted changes in taxonomic and functional diversity of tropical fish communities after habitat degradation. *Ecol. Appl.* **20**, 1512-1522 (2010).
